# Prediction of Deoxynivalenol Contamination in Wheat via Infrared Attenuated Total Reflection Spectroscopy and Multivariate Data Analysis

**DOI:** 10.1021/acsfoodscitech.3c00674

**Published:** 2024-03-25

**Authors:** Polina Fomina, Antoni Femenias, Valeria Tafintseva, Stephan Freitag, Michael Sulyok, Miriam Aledda, Achim Kohler, Rudolf Krska, Boris Mizaikoff

**Affiliations:** † Institute of Analytical and Bioanalytical Chemistry, Ulm University, Albert-Einstein-Allee 11, 89075 Ulm, Germany; ‡ Faculty of Science and Technology, Norwegian University of Life Sciences, Drøbakveien 31, 1432 Ås, Norway; § University of Natural Resources and Life Sciences, Vienna, Department of Agrobiotechnology IFA-Tulln, Institute of Bioanalytics and Agro-Metabolomics, Konrad Lorenzstr. 20, A-3430 Tulln, Austria; ∥ Institute for Global Food Security, School of Biological Sciences, Queen’s University Belfast, 19 Chlorine Gardens, BT9 5DL Belfast, Northern Ireland; ⊥ Hahn-Schickard, Sedanstraße 14, 89077 Ulm, Germany

**Keywords:** attenuated total reflection, ATR, infrared spectroscopy, Fourier transform infrared spectroscopy, FTIR, deoxynivalenol, DON, fungal infection, mycotoxins, wheat, sparse partial discriminant least-squares analysis, SPLS-DA

## Abstract

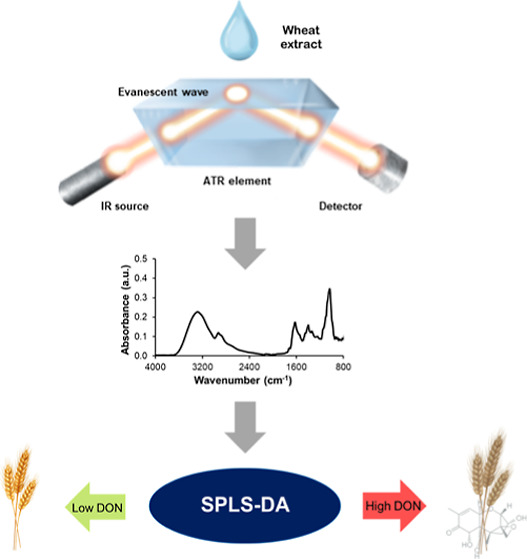

The climate crisis further exacerbates the challenges for food production. For instance, the increasingly unpredictable growth of fungal species in the field can lead to an unprecedented high prevalence of several mycotoxins, including the most important toxic secondary metabolite produced by *Fusarium* spp., i.e., deoxynivalenol (DON). The presence of DON in crops may cause health problems in the population and livestock. Hence, there is a demand for advanced strategies facilitating the detection of DON contamination in cereal-based products. To address this need, we introduce infrared attenuated total reflection (IR-ATR) spectroscopy combined with advanced data modeling routines and optimized sample preparation protocols. In this study, we address the limited exploration of wheat commodities to date via IR-ATR spectroscopy. The focus of this study was optimizing the extraction protocol for wheat by testing various solvents aligned with a greener and more sustainable analytical approach. The employed chemometric method, i.e., sparse partial least-squares discriminant analysis, not only facilitated establishing robust classification models capable of discriminating between high vs low DON-contaminated samples adhering to the EU regulatory limit of 1250 μg/kg but also provided valuable insights into the relevant parameters shaping these models.

## 1 Introduction

In the face of climate change (CC) (e.g., global warming and rainfall), food security is threatened.^1,2^ For instance, CC effects such as increases in temperatures and alterations in precipitation patterns render the control of the growth of fungal species in agricultural fields increasingly challenging.^3^ This may also lead to shifts in the occurrence of toxic secondary metabolites produced by these fungi—so-called mycotoxins—that are harmful to humans and livestock.^4^ Mycotoxin prevalence in crops is dependent on environmental and biological factors, pre- and post-harvest practices, and storage conditions. Mycotoxin contamination leads to substantial economic losses for farmers, producers, and processors of crops. For wheat alone, 1.2–2.4 billion euros are lost annually in Europe.^5^ In addition, serious health problems may occur due to the consumption of mycotoxins.^6^

Deoxynivalenol (DON), formerly also known as vomitoxin, is among the most prevalent mycotoxins in wheat produced primarily by *Fusarium graminearum* and *Fusarium culmorum*.^7^ The analysis of recent data on food grains for the years 2010–2015 provided by the European Food Safety Authority (EFSA) and Biomin Inc. revealed a DON prevalence of 60%. Overconsumption of such *Fusarium* mycotoxins causes vomiting, feed refusal, and reduced absorption of the nutrient in animals.^8^ The acute exposure to DON may cause diarrhea, headache, dizziness, fever, and bloody stool in humans.^9^ Due to the regular exposure of humans and animals, DON has become a global food safety issue and prompted the authorities to set guidelines on the maximum allowed levels of DON in wheat and other grain crops.^10,11^ In 2006, the European Commission (EU) established the DON limit for unprocessed common wheat at 1250 μg/kg.^12^

Due to its toxicity and economic impact, there is a significant demand for advanced strategies to detect DON contamination in cereal-based products. This is further underlined by the fact that cereals and their derived products are among the main components of the diet worldwide. According to the Food and Agricultural Organization of the United Nations (FAO), cereals represent 34% of the total diet.^13^ Analytical methods for the detection of mycotoxins and, in particular, DON usually include sophisticated instrumental analytical methods such as, but not limited to, liquid chromatography coupled to mass spectrometry (LC–MS/MS), which is the gold standard method for the sensitive and simultaneous detection of multiple mycotoxins.^14,15^ Immunoanalytical approaches including enzyme-linked immunosorbent assays (ELISA) or lateral flow devices (LFD), on the other hand, are used for rapid screening of single toxins or toxin groups.^16^ The main drawbacks of LC–MS include high analytical costs, extensive sample preparation, and limited in-field capabilities, preventing rapid (on-site) mycotoxin monitoring. Conversely, ELISA or LFD suffers from cross-reactivities and requires organic extraction solvents.^17^ Therefore, there is a demand for methods that facilitate rapid, low-cost, and sustainable analysis of contaminants to control the quality of products in the food and feed industry. Mid-infrared (MIR) spectroscopy appears to be a viable solution to address this demand given its inherent molecular selectivity along with little-to-no sample preparation, rendering MIR spectroscopy a rapid and green analytical method.

MIR spectroscopy and other spectroscopic techniques have been used to screen for mycotoxins in corn, nuts, fruits, etc.^18−20^ Infrared (IR) spectroscopy enables the rapid acquisition of absorption spectra with limited or even without sample preparation and/or pretreatment, and facilitates the noninvasive analysis of samples in any state of aggregation. In the MIR regime, fundamental vibrational modes are excited providing detailed qualitative and quantitative molecular information.^21^ Besides the advantages of MIR spectroscopy, fundamental vibrations are pronounced spectral features, facilitating molecular discrimination. Attenuated total reflection (ATR) is a sampling technique for solid, semisolid, or liquid samples deposited directly at the surface of an ATR waveguide for recording evanescent field absorption spectra, whereby additional sample manipulation is not needed.^22^ Cleaning of the waveguide surface after each sample is the only procedure that is required. In combination with appropriate multivariate data analysis strategies, relevant information on the molecular composition and/or the presence of contaminants can be extracted even from complex food or feed matrices.^23^ In addition, IR spectroscopic techniques require a minimum of consumables or reagents; hence, IR-ATR spectroscopy is considered a “green” method.^24^ Recent advancements in IR photonics have even demonstrated portable hand-held analyzers and sensors.^25^

The present study focused on the capabilities of IR-ATR spectroscopy for monitoring DON contamination in wheat due to the to date rather limited analysis of the commodity via this analytical technique. IR-ATR spectroscopy has already been successfully utilized for mycotoxin analysis in maize. For instance, Kos et al. pioneered the usage of IR-ATR for *Fusarium* toxin prediction.^26,27^ However, compared to other sampling techniques, IR-ATR requires minimal amounts of sample. Kos and co-workers recorded several subspectra per sample to enhance the representativeness of the sample. Follow-up research of Kos et al. has used a sieving step to control the particles size counteracting reproducibility issues.^28^ In a more recent study, Kos et al. aimed at the classification of contaminated samples at a defined threshold.^29^ Sieger et al. was using extracts from ground material combined with laser-based MIR spectroscopy.^30^ Femenias et al. streamlined this approach to screen for DON in maize by IR-ATR spectroscopy. The sample extracts were optimized to further enhance the representativity of the recorded spectra. The classification of maize into high vs low DON-contaminated samples (EU-regulatory limit for DON in corn is 1750 μg/kg)^12^ was achieved with high accuracy using water and methanol/water (70:30) mixtures as solvents for DON extraction. Sparse partial least-squares discriminant analysis (SPLS-DA) models with an accuracy of cross-validation equal to 86.7% were shown for DON extracts using water as a solvent and 90.8% for DON extracts using methanol/water (70:30).^31^ SPLS-DA analysis was preferred over PLS-DA as it is a variable selection method that allows building sparse classification models. Sparse models reduce overfitting, which is a known problem in chemometrics and spectroscopy due to the number of potentially correlated variables vs comparatively few samples in the data set. In addition, sparse models are easier to interpret as only a few variables are present in the regression coefficient.^32^

The number of studies reporting the determination of DON in wheat via IR spectroscopy is limited.^33^ Hence, the current study focuses on the optimization of DON analysis in wheat by testing extraction solvents establishing an efficient, reliable, and eco-friendly analytical methodology. We hypothesize that using a variety of extraction solvents with different polarities facilitates the extraction of different component ratios (i.e., proteins vs lipids vs carbohydrates), which in turn affects DON prediction in wheat.

## 2 Materials and Methods

### 2.1 Sample Preparation

**Figure 1:**
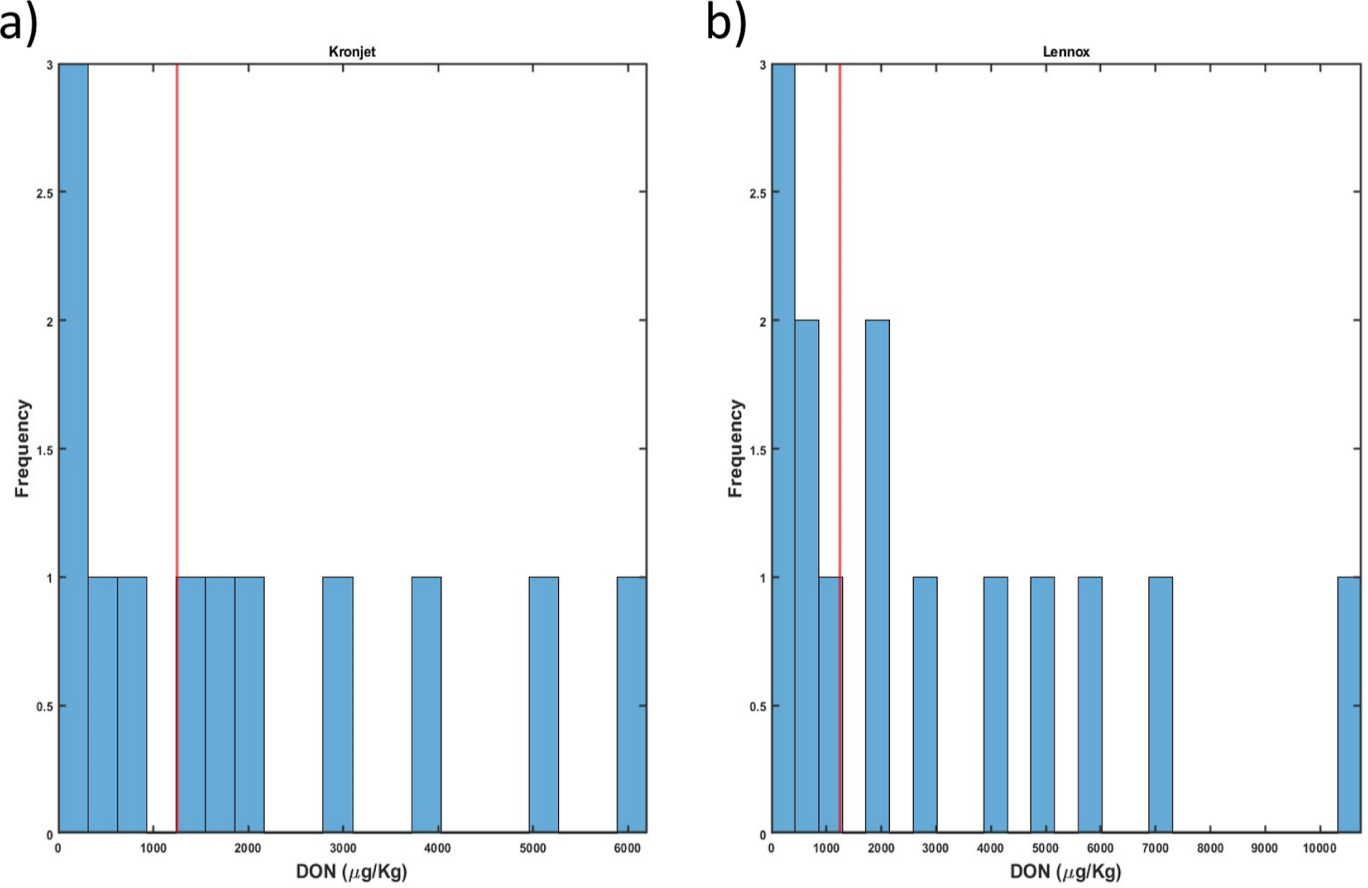
Histogram of DON concentrations in (a) Kronjet wheat and (b) Lennox wheat. Red line is the threshold value of 1250 μg/kg.

Two types of wheat varieties (Lennox and Kronjet) with different harvesting seasons (facultative and spring, respectively) were supplied by Lantmännen SW Seed AB (Switzerland) and KWS Lochow GmbH (Germany). From each variety, wheat with a high concentration of DON was obtained by inoculating *F. graminearum* and *F. culmorum* using kernel-spawn and spraying methods in the greenhouse during the winter of 2021/2022. Briefly, *F. graminearum* colonized wheat kernels by distributing the fungi on the soil surface between the wheat plants about 3 weeks before anthesis (ca. 15 gr/m^2^). Perithecia produced on the surface eject ascospores in the air, which infect wheat ears. Alternatively, a suspension of 50,000 conidia/mL of *F. graminearum* and *F. culmorum* was sprayed (100 mL/m^2^) during the flowering time onto the wheat varieties. Low-contaminated wheat was obtained by 2021 field trials. Blank and contaminated materials were blended to obtain increasing DON concentrations, as follows. For the Lennox variety, a sample contaminated with 10,600 μg/kg (obtained by greenhouse trials) was blended with a blank sample, from the field, producing a set with DON concentrations of 100, 200, 500, 750, 1250, 2000, 3000, 4000, 5000, 6000, 7000, and 10,600 μg/kg. For the Kronjet variety, a sample (from the greenhouse) contaminated with 6020 μg/kg was mixed with a blank sample (from the field), producing DON contaminations of 100, 200, 500, 750, 1250, 2000, 3000, 4000, 5000, and 6020 μg/kg. Figure 1 demonstrates the DON concentration distribution within the Kronjet and Lennox samples. After blending the samples, the entire kernels were ground into a fine powder using a mill (Perten LabMill 3610, PerkinElmer). The resulting powder was stored at a temperature of 4 °C until it was extracted and analyzed.

Before extraction, the wheat powder was shaken to homogenize the mycotoxin content, and 2 g was transferred into a 15 mL tube, weighting them at the precision balance (Sartorius GmbH, Gottingen, Germany). Three aqua-based solvent systems were prepared to extract DON from the samples (v/v, shown in parentheses): water (100), ethanol/water (30:70), and methanol/water (30:70). The same sample set was extracted in parallel with the three solvent systems by adding 8 mL of the solvent to the solid wheat powder and enhancing the solid–liquid phase transfer by shaking the tube for 60 min at 120 rpm on a Rocking platform (VWR, UK). The solid–liquid mixtures were centrifuged for 2 min at 6000 rpm, and 2 mL of the liquid supernatants were transferred to a 2 mL Eppendorf tube (Eppendorf AG, Hamburg, Germany). After extraction, the samples were analyzed via ATR-FTIR spectroscopy.

### 2.2 Reference Analysis

Milled wheat samples were extracted in 50 mL centrifuge tubes (Sarstedt, Nürnbrecht, Germany) with a solid/liquid ratio (1:4, m/v) by mixing 5 g of solid material with the extraction solvent (acetonitrile/water/acetic acid 79:20:1, v/v/v) in a GFL 3017 rotary shaker for 90 min (GFL, Burgwedel, Germany). Thereafter, 500 μL of the extract were transferred to autosampler vials and diluted (1:1, v/v) with the extraction solvent (acetonitrile/water/acetic acid 79:20:1, v/v/v). After proper homogenization, 5 μL of the extract was analyzed with LC–MS/MS with a QTrap 5500 LC–MS/MS System (Applied Biosystems, USA) provided with TurboIonSpray electrospray ionization (ESI) source and 1290 Series high-performance liquid chromatography (HPLC) (Agilent, Germany). Detailed information on the instrumental conditions, calibration, and validation of the reference method (including proficiency testing result) is given in Sulyok et al.^34^

### 2.3 Infrared Spectroscopy

An FTIR spectrometer (Alpha II, Bruker Optics GmbH, Germany) was utilized for the spectroscopic measurements. The device is equipped with a single-bounce diamond ATR assembly (Platinum ATR, Bruker Optics GmbH, Germany) and a room-temperature operated RT-DLaTGS detector. The Opus 8.1. software package (Bruker Optics GmbH, Germany) was used to collect spectra over the entire MIR range (4000–400 cm^–1^). For each sample and background measurement, 128 scans were averaged at a spectral resolution of 4 cm^–1^. Three technical repetitions were recorded for every sample. For each extract, a 5 μL aliquot was placed on the surface of the ATR crystal, ensuring complete coverage, and allowed to evaporate for 10 min.

### 2.4 Data Analysis

FTIR spectra were preprocessed as follows: (1) second derivative using Savitzky–Golay algorithm with a window size of 13 and a second-order polynomial; (2) selection of spectral region of interest (SROI): 1800–900 cm^–1^; (3) normalization by extended multivariate signal correction (EMSC) with linear and quadratic terms.^35^ The determination of the SROI was based on the most significant bands of DON (Table 1), which are located in the “fingerprint” range of the MIR spectrum, namely, 1800–900 cm^–1^.^33,36,37^

**Table 1:** Assignment of the Wheat and DON Bands in the IR-ATR Spectra^33,36,37,42^

wavenumber, cm^–1^	band assignment
	wheat bands
1745	C=O ester stretching, lipids, carbohydrates
1642	amide I (C=O stretching): proteins, pectins
1540	amide II (C–N stretching, N–H bending): proteins
1446	C–H: cell wall polysaccharides, alcohols and carboxyl acids
1239	amide III (C–N and N–H bending): proteins
DON bands	
1685	C=O (carbonyl group)
1167	C–O–C antisymmetric stretching
1069	RCH–OH stretching
956	C–O (epoxide ring) stretching

Data mining and outlier detection were performed by conducting principal component analysis (PCA), whereas SPLS-DA was used for the classification. Both approaches are multivariate dimensionality reduction tools; in addition, SPLS-DA enables feature/variable selection and classification.^38−40^ SPLS-DA makes a sparsity assumption, i.e., only a small number of variables are responsible for driving the effect under study.^32,41^ The SPLS algorithm penalizes the loading weight vectors according to a parameter called the degree of sparsity, which determines the number of zeros for a given loading. For each PLS component, we chose 99% sparsity; i.e., for each PLS component, 99% of the variables were penalized and thus set to zero, and only 1% of the variables were used to build the model.

To establish the classes (high vs low DON contamination), the threshold was set at 1250 μg/kg according to the EU regulations for DON levels in wheat.^12^ Thereby, we considered samples with a DON concentration >1250 μg/kg as high contaminated and those with a DON concentration ≤1250 μg/kg as low contaminated. All the models were built using leave-one-out cross validation (CV), where three technical repetitions of the same sample were taken out together at each step of the CV. Classification accuracy of the CV (Acc_cv_) was used as a metric of the model performance since it represents the ratio of correct predictions to the total number of predictions. Regression coefficients, the number of latent variables (LVs), specificity (the model’s ability to predict true negatives in each available category), and sensitivity (the model’s ability to predict true positives in each available category) were used to explore the models in more detail. Data analyses were performed by standard algorithms and algorithms developed in-house in MATLAB, R2022a (Mathworks Inc., Natick, Massachusetts).

## 3 Results

### 3.1 IR Spectra of Wheat Extracts

**Figure 2:**
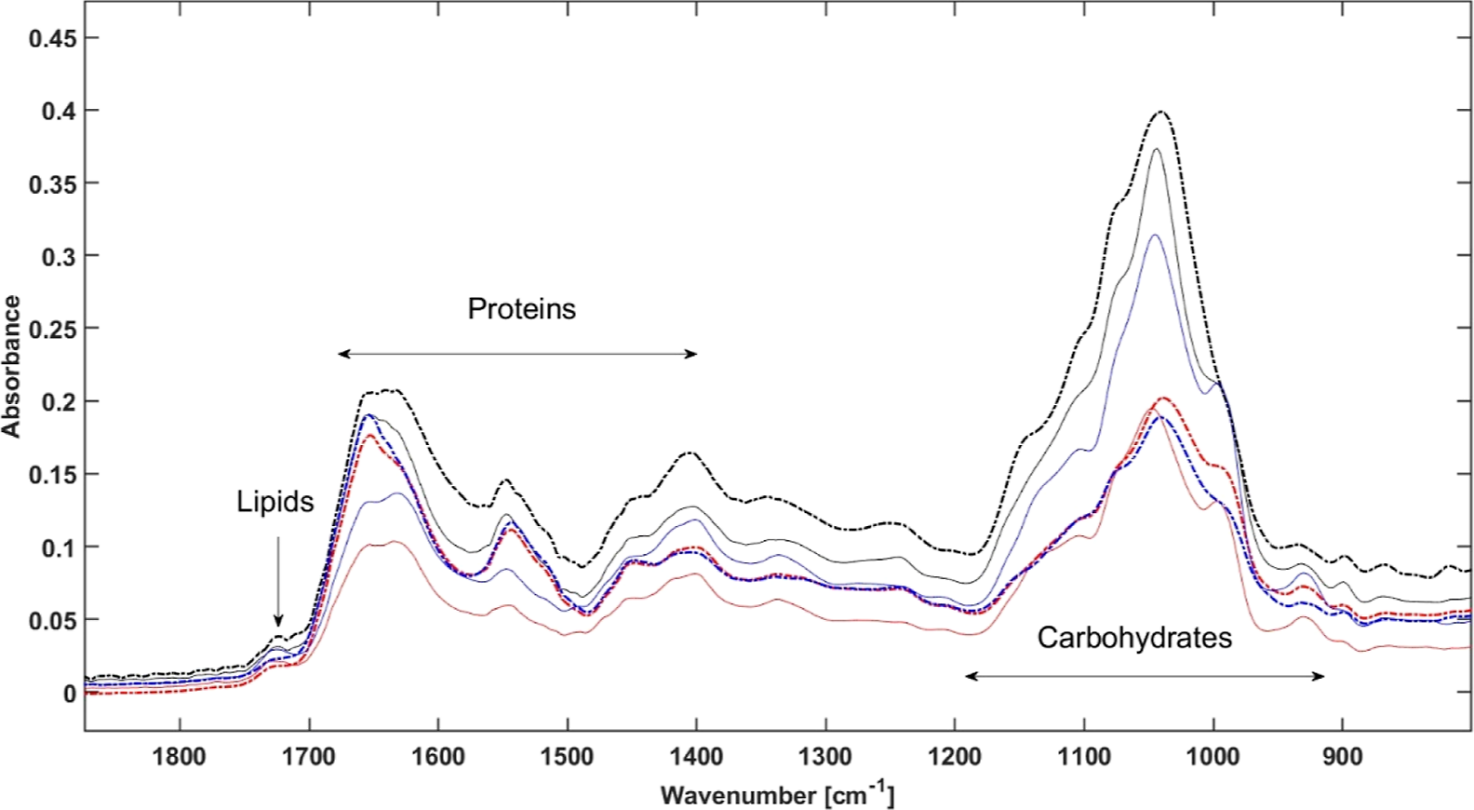
Baseline corrected spectra of high (10,600 μg/kg; dotted lines) and low [below LOD (1.2 μg/kg) of the reference method; continuous lines] DON wheat (Lennox) extracts with water (black), ethanol 30% (red), and methanol 30% (blue) in the range 1800–900 cm^–1^.

Figure 2 illustrates baseline corrected spectra of the wheat extracts with high (10,600 μg/kg) and low [below LOD (1.2 μg/kg) of the reference method] DON concentrations after solvents evaporation. These spectra of the dried extracts do not show any pronounced characteristic bands of DON, which are listed in Table 1, apparently due to the strong matrix contribution. However, we can observe changes in the intensity levels for the high- and low-DON-contaminated extracts (Figure 2), what means that the approach of MIR-ATR can be considered as an “indirect” analysis of DON. Based on the existing literature, it is evident that the spectra can be partitioned into multiple segments, including the lipid range, protein range, and carbohydrate range, as it is shown in Figure 2.^42^ This implies that these particular substances predominantly account for the spectral features within their respective ranges. Table 1 lists the characteristic bands of wheat. In addition, it is worth noting that the spectra (Figure 2) after the evaporation of the different solvents exhibit similarities in the patterns, but they are not identical. This supports the hypothesis that the different solvents allow extracting different components in different ratios, hence affecting the performance of the assay.

### 3.2 PCA Analysis

**Figure 3:**
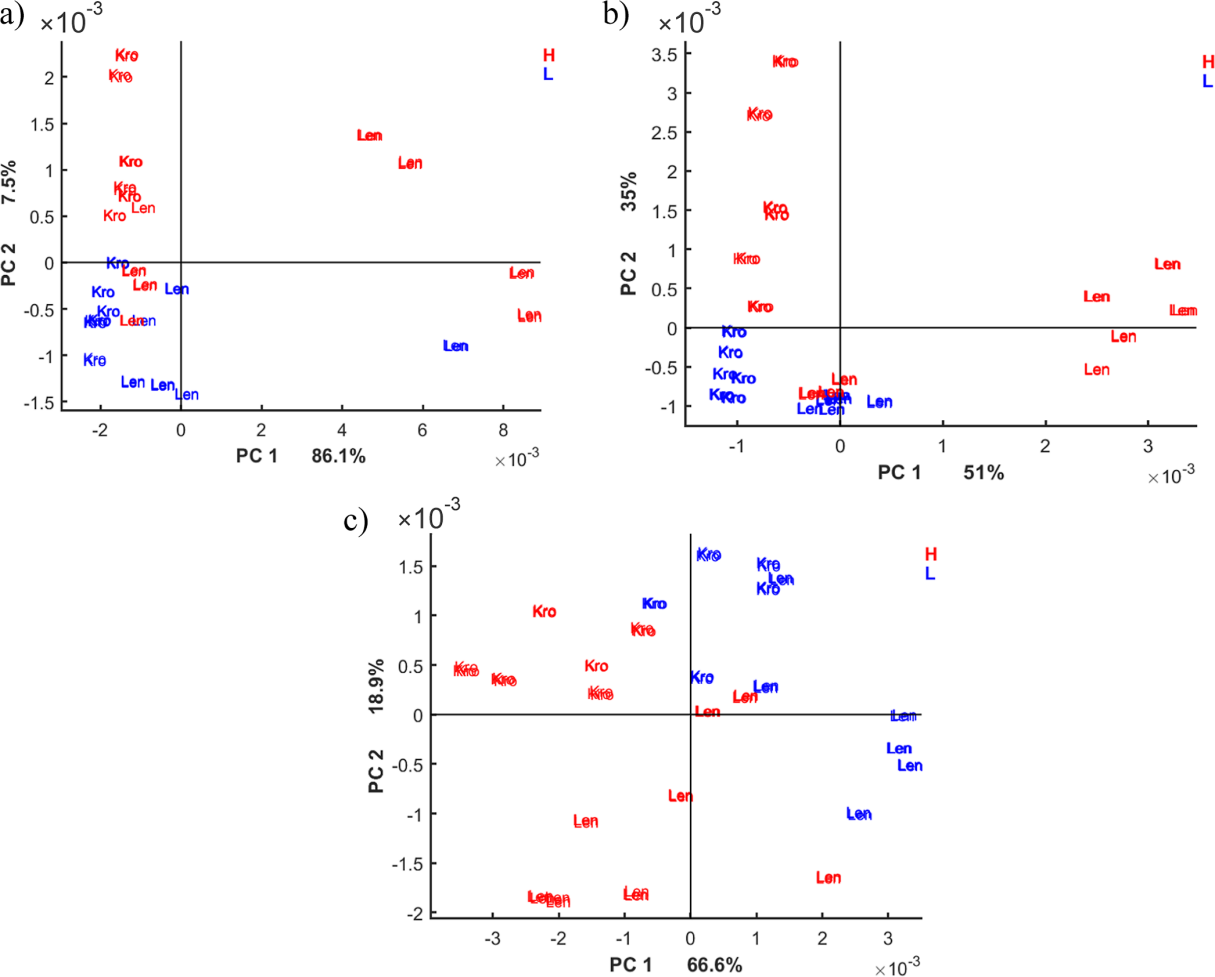
PCA score plots for the first two PCs of the wheat extracts by different solvents (a) methanol/water (30:70), (b) ethanol/water (30:70), and (c) water (100). High-contaminated samples are shown in red and low-contaminated samples in blue. Kro—samples were obtained from Kronjet wheat; Len—samples were obtained from Lennox wheat.

PCA was applied to evaluate the recorded MIR spectra of the wheat extracts in order to investigate the patterns and behavior of the data. It is crucial to examine how the model performs based on the extraction solvent used to optimize the extraction procedure and choose the most effective one. For the PCA analysis, samples were labeled by the different types of wheat (Kronjet—Kro and Lennox—Len) and colored by the DON contamination level (Low—L and High—H). The threshold between the high and low contaminations was set at 1250 μg/kg, whereby the actual sample concentration was verified via LC–MS/MS analysis (see 2.2). Figure 3 displays the PCA score plots for the first two principal components (PCs) of the models built by using the spectra of the different solvents.

PCA analysis indicates clustering according to the wheat type, which is particularly pronounced for the ethanol/water (30:70) (along PC 1) and water (100) (along PC 2) models. On the other hand, the PCA score plots display a tendency for the clustering according to the DON values, which can be clearly noted for the water (100) (along PC 1) model. For the ethanol/water (30:70) and methanol/water (30:70) models, the tendency for the clustering could be observed within the Kronjet wheat type (along PC 2) and rather difficult within the Lenox wheat type.

**Figure 4:**
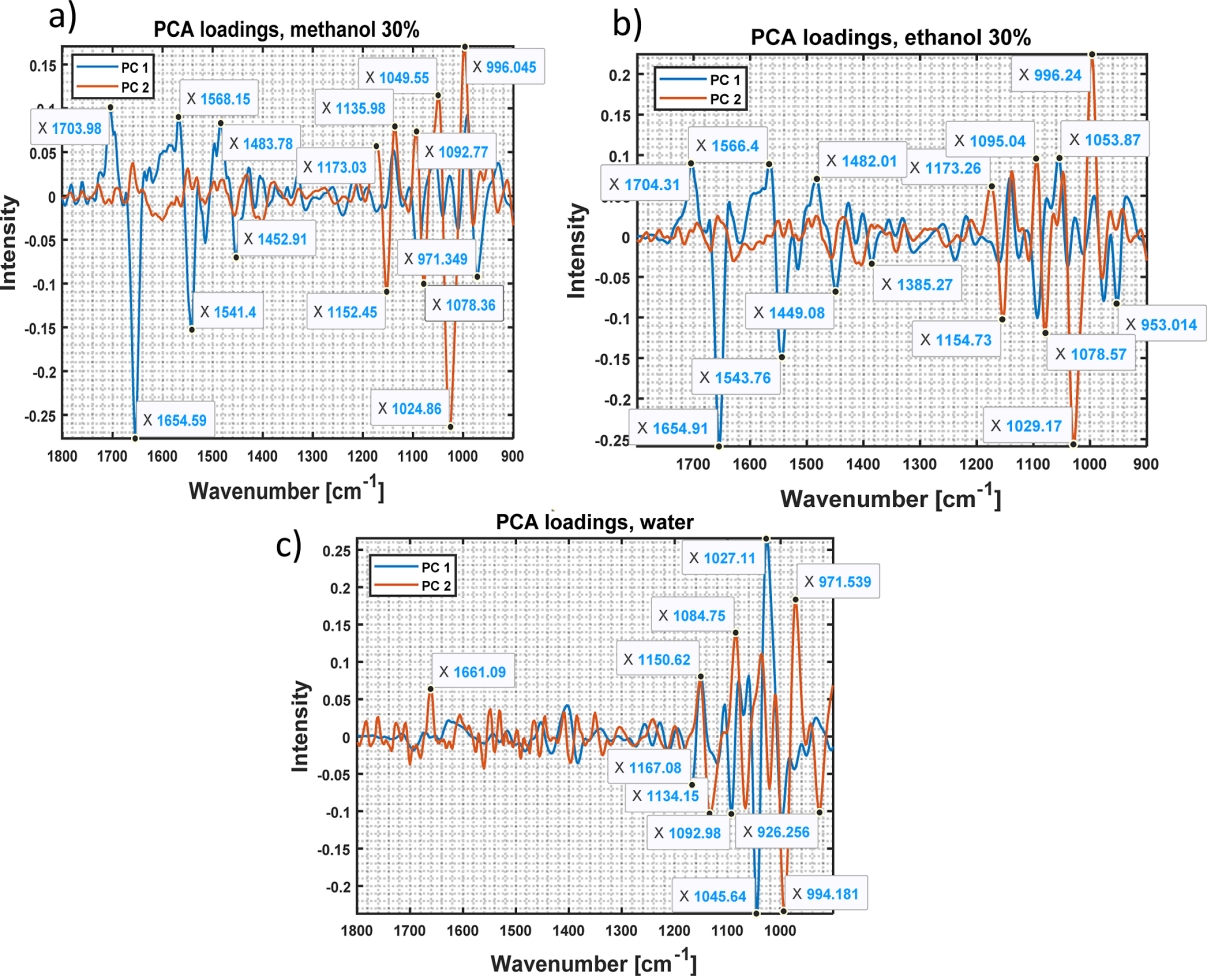
PCA loading plot for the first two PCs of different solvents: (a) methanol/water (30:70), (b) ethanol/water (30:70), and (c) water (100). Blue line—PC 1 loadings; orange line—PC 2 loadings.

To gain more insights from the PCA models, it is worthwhile to investigate the PCA loading plots (Figure 4). Comparing the loading plots for the different solvents, we can observe that the loadings of the methanol/water (30:70) and ethanol/water (30:70) extracts share similarities which is reasonable due to the similar relative polarity of 0.627 and 0.634, respectively.^43^ Specifically, PCA models for both solvents present a large spread in intensities observed in the range of 1750–1450 cm^–1^ for PC 1, which mainly consists of protein vibrations, as well as a large spread in intensities observed in the range of 1150–1000 cm^–1^ for PC 2, which mainly consists of carbohydrate vibrations. Figure 3a,b demonstrates that PC 1 in both models mainly corresponds to the clustering according to the wheat type, and both PCs 1 and 2 contribute to the clustering according to the DON levels. These results indicate that the clustering according to the wheat type may be caused by the distinct protein content of the samples, and the difference in DON contamination is caused by the variations in both the protein and carbohydrate composition of the samples.

The loading plot for the pure water model is not identical to the other loading plots (Figure 4c). The largest difference for both PCs is observed mainly in the spectral range of carbohydrates. In contrast to other models, the trend toward clustering is reversed in this model. PC 1 is mainly responsible for the clustering according to the DON concentrations and PC 2 according to the wheat type.

The PCA analysis revealed no pronounced outliers, and all samples were therefore used for the classification models.

### 3.3 SPLS-DA Analysis

The binary classification of wheat samples into contaminated and noncontaminated groups was carried out using SPLS-DA. The threshold for the models was set again at the EU-limit of 1250 μg/kg. Due to the limited number of samples, the models were established by utilizing cross-validation. The results are summarized in Table 2 and visualized by the confusion matrices in the Supporting Information of this study (S 1). The superior classification into high and low DON was obtained for the water solvent, with a cross-validation classification accuracy of 91%. Slightly inferior perform ethanol/water (30:70) and methanol/water (30:70) solvents and correspond to cross-validation classification accuracy of 88.5 and 84.6%, respectively. The number of LVs in a PLS-DA model illustrates the complexity of the classification models: how many components are required to separate the groups. As can be seen in these cases (see Table 2), the models have low complexity: only 1 LV for the ethanol/water extracts and 4 LVs for the methanol/water (30:70) and water (100) extracts. The specificity values of all the models exceed 90%, while the sensitivity of the ethanol/water (30:70) and methanol/water (30:70) models is 79 and 86% in the case of water (100).

**Table 2:** SPLS-DA Classification Results of the High- and Low-DON-Contaminated Wheat Extracts with Different Solvents

solvent	Acc_cv_, %	#LVs	specificity, %	sensitivity, %
methanol/water (30:70)	84.6	4	92	79
ethanol/water (30:70)	88.5	1	100	79
water (100)	91.0	4	97	86

**Figure 5:**
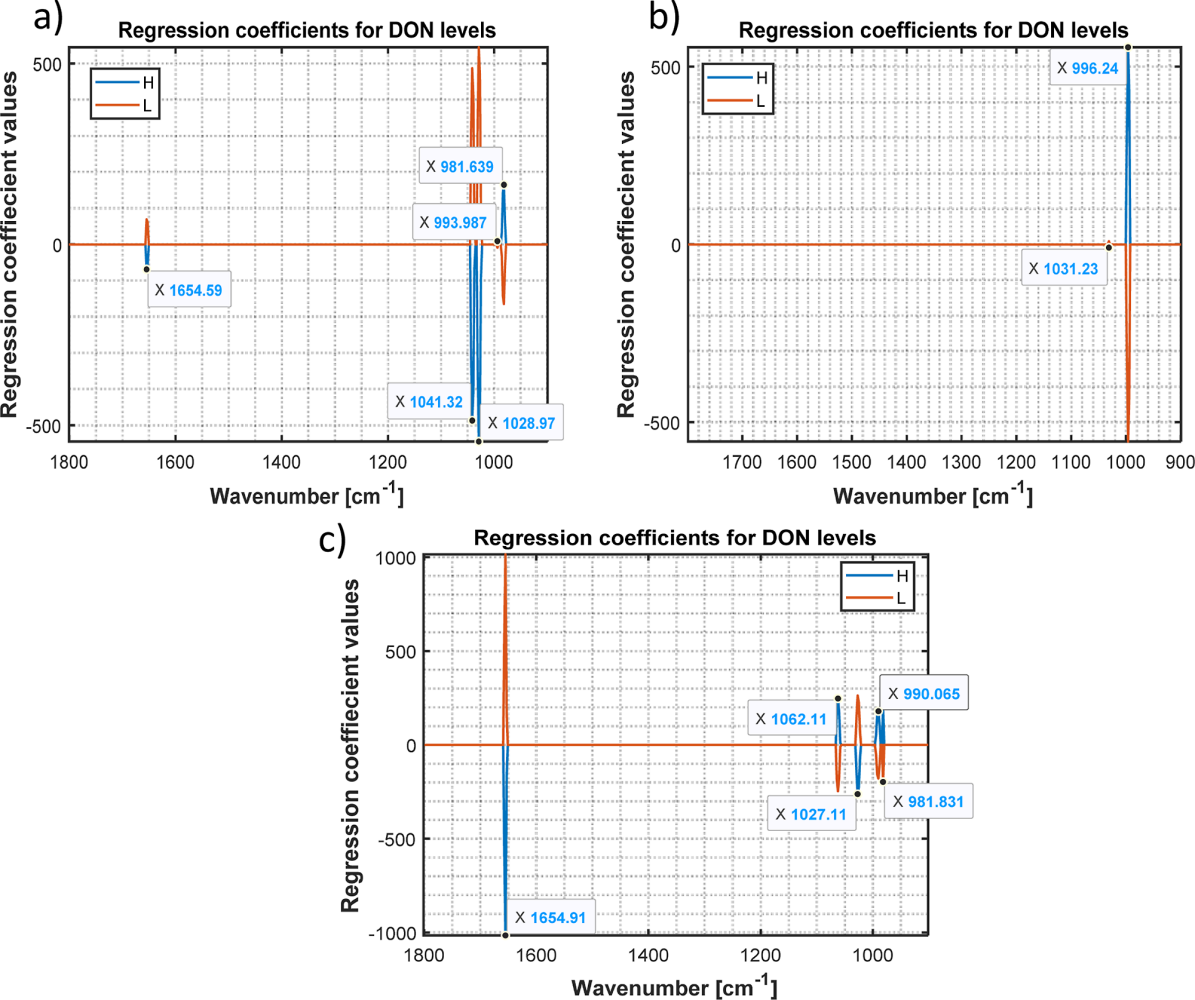
SPLS-DA regression coefficients of the models obtained from the different solvents: (a) methanol/water (30:70), (b) ethanol/water (30:70), and (c) water (100). Blue lines correspond to the high-DON-contaminated samples (DON > 1250 μg/kg), and orange lines correspond to the low-DON-contaminated samples (DON < 1250 μg/kg).

The choice of classification algorithm was made to simplify the interpretation of the results. Consequently, the regression coefficients were visualized and analyzed (see Figure 5a–c). It is evident that the classification in all models is based on a few bands in the protein and carbohydrate spectral regions. More complex models of methanol/water (30:70) and water extracts are based on the more spectral variables including one amide I band at 1654 cm^–1^ and several bands in the carbohydrate region around 1027–1029 cm^–1^ and one band at 981 cm^–1^, which are consistently present in both models (Figure 4a,c). In contrast, the ethanol/water (30:70) model, being very simple, is based on one band in the carbohydrate range at 994 cm^–1^ (Figure 4b). These results confirm the nature of the discrimination between contaminated and noncontaminated wheat samples as indirect detection of the matrix change.

## 4 Discussion

The current study examined the potential of IR-ATR spectroscopy for differentiating between high and low DON contamination levels in wheat, investigating extracts, and using a variety of samples with different DON levels obtained by blending contaminated and blank material. The threshold level selected follows the EU requirements regarding the permissible levels in common wheat (1250 μg/kg).^12^

The characteristic peaks of DON are not clearly evident in the IR spectra of the extracts due to matrix interferences (Figure 2 and Table 1). Therefore, we assume that IR-spectroscopic approaches are indirect analysis methods that rely on associated changes in the matrix due to fungal infection and fungal growth. In Femenias et al., the authors showed comparable findings.^31^ This assumption allows us to interpret further data analysis outcomes.

The findings of the unsupervised algorithms (PCA) demonstrate clustering according to the different wheat types (Lennox and Kronjet). However, we expect that it does not interfere with the DON analysis, since some models show likewise a trend toward clustering according to DON contamination (high and low), in particular, the water (100) model. Again, in the study of Femenias et al., it was observed that the samples cluster not only according to the DON concentrations but also according to the other parameters, i.e., the type of contamination (naturally vs inoculated). No evidence was found that this clustering influences the classification according to the DON levels.^31^

From the obtained SPLS-DA results (Table 2), it is evident that all the models show a high classification accuracy of cross-validation (Acc_cv_ > 0.840). The prediction models exhibit high values of sensitivity and specificity, suggesting a balanced classification of both classes: low and high DON (Table 2). The best performing model was achieved using pure water as an extraction solvent, while methanol/water (30:70) and ethanol/water (30:70) demonstrate slightly worse performance (see Table 2). Selecting pure water over other mixtures with organic solvents offers benefits including being green, eco-friendly, nontoxic, and nonflammable, which makes the extraction a sustainable process.^44^ This type of eco-friendly sample preparation procedure ensures that the entire analysis workflow is safe for the environment and human operators. It is noteworthy that during the investigation of DON-contaminated maize samples in other studies, methanolic solvents exhibited superior performance compared to aqueous solutions. This could be attributed to the distinct chemical composition of maize and wheat and/or the nature of the fungal contamination: the type of fungal strains grown, which metabolites they produce, and how they degrade the food matrix.^31^

The high classification accuracy of the models achieved in the present study is indeed consistent with the literature. Several other studies that applied IR-ATR spectroscopy for mycotoxins analysis in cereals or other matrices demonstrate Acc_cv_ > 90%. For instance, the study of De Girolamo et al. could distinguish high and low levels of ochratoxin A-contaminated wheat samples with a cutoff limit at 2 μg/kg and Acc_cv_ = 94%.^45^ In the study by Kaya-Celiker et al., it was shown that “highly moldy” (>1200 μg/kg), “moldy” (between 20 and 1200 μg/kg), and “acceptable” (<20 μg/kg) peanuts contaminated with aflatoxin can be differentiated using the MIR-ATR technique. The obtained model provided a Acc_cv_ of 98.5% with only one misclassified sample over 164 samples.^46^

The SPLS-DA algorithm combining variable selection and PLS provides the models with very sparse regression coefficients. Only a few spectral features were required to provide good classification of DON contamination in wheat. Such sparse models have also been demonstrated to be able to discriminate between different types of samples. For instance, the study of Zimmerman demonstrates the classification of the genera of pollen samples using just one band.^47^ Another recent study has shown the potential of the sparse data analysis to predict milk fatty acid composition.^48^ This suggests that instead of broadband spectral measurements, it may indeed be feasible to analyze only few specific spectral bands and still obtain reliable discrimination models. In the future, compact monochromatic light sources such as quantum and interband cascade lasers may be utilized for DON screening instead of broadband sources. This allows for a significant miniaturization of the device, increased portability, and potentially lower price. However, further studies are required to optimize the variable selection, such that it is representative for DON contamination in wheat.

Another noteworthy observation is that suitable classification models for DON analysis do not require collecting data from exceedingly large quantities of contaminated and noncontaminated cereal samples from the field, as done during similar studies, e.g., by Kos et al. or by Öner et al.^20,26,49^ A small quantity of highly contaminated and blank samples is sufficient to conduct blending and produce samples at varying concentrations, as was achieved in the present study. This approach may radically simplify the analysis workflow, thereby reducing time and costs for analytical laboratories. In contrast to the fundamental feasibility shown herein, future research will conduct the validation using external data sets to prove the reliability of the models.

The overall findings of the present study suggest that the classification of wheat samples according to high vs low DON contamination is particularly suitable via IR-ATR spectroscopy taking advantage of the grain chemical composition, which is influenced by a variety of parameters including the *Fusarium* species colonizing the cereal. Optimized extraction procedures are based on pure water as the extraction solvent and clearly enhance the predictive abilities of the classification models. As shown herein, extracts with pure water indeed yielded the best classification performance of the associated multivariate models. In general, IR-ATR spectroscopy combined with machine learning algorithms demonstrated substantial potential for DON screening that could readily be transitioned from the laboratory to field applications by miniaturizing the IR photonic sensing devices.

## Supporting Information Available

Confusion matrices of the results of the SPLS-DA classification for wheat samples in two groups (PDF)

## Abbreviations

ATR: attenuated total reflection
IR: infrared
MIR: mid-infrared
DON: deoxynivalenol
FTIR: Fourier transform infrared spectroscopy
EMSC: extended multivariate signal correction
SPLS-DA: sparse partial discriminant least-squares analysis
PCA: principal component analysis
PC: principal component
LV: latent variable
EU: European Union
CC: climate crisis
FAO: Food and Agricultural Organization of the United Nations
Acc_cv_: accuracy of cross-validation
CV: cross validation
LC-MS/MS: liquid chromatography coupled to mass spectrometry
SROI: spectral region of interest
ELISA: enzyme-linked immunosorbent assays
LFD: lateral flow devices
ESI: electrospray ionization
HPLC: high-performance liquid chromatography
