# Supplementary material for: Prediction of Deoxynivalenol Contamination in Wheat via Infrared Attenuated Total Reflection Spectroscopy and Multivariate Data Analysis
Source: ACS Food Sci Technol. 2024 Mar 25;4(4):895–904. doi: 10.1021/acsfoodscitech.3c00674 (PMC11037394; doi:10.1021/acsfoodscitech.3c00674)
Supplement: Supplementary file 1 — fs3c00674_si_001.pdf [file fs3c00674_si_001.pdf]

Prediction of Deoxynivalenol Contamination in Wheat via Infrared Attenuated Total Reflection Spectroscopy and Multivariate Data Analysis

Polina Fomina[1], Antoni Femenias[1], Valeria Tafintseva[2], Stephan Freitag[3], Michael Sulyok[3], Miriam Aledda[2], Achim Kohler[2], Rudolf Krska[3,4] and Boris Mizaikoff[1,5*]

[1]*Institute of Analytical and Bioanalytical Chemistry, Ulm University, Albert-Einstein-Allee 11, 89075 Ulm, Germany*

[2]*Faculty of Science and Technology, Norwegian University of Life Sciences, Drøbakveien 31 ,1432 Ås, Norway*

[3]*University of Natural Resources and Life Sciences, Vienna, Department of Agrobiotechnology IFA-Tulln, Institute of Bioanalytics and Agro-Metabolomics, Konrad Lorenzstr. 20, A-3430 Tulln, Austria*

[4]*Institute for Global Food Security, School of Biological Sciences, Queen's University Belfast, 19 Chlorine Gardens Belfast BT9 5DL, Northern Ireland*

[5]*Hahn-Schickard, Sedanstraße 14, 89077 Ulm, Germany*

**Corresponding author: boris.mizaikoff@uni-ulm.de*

Supplementary materials

Number of pages: 2

Number of figures: 1

Number of tables: 0

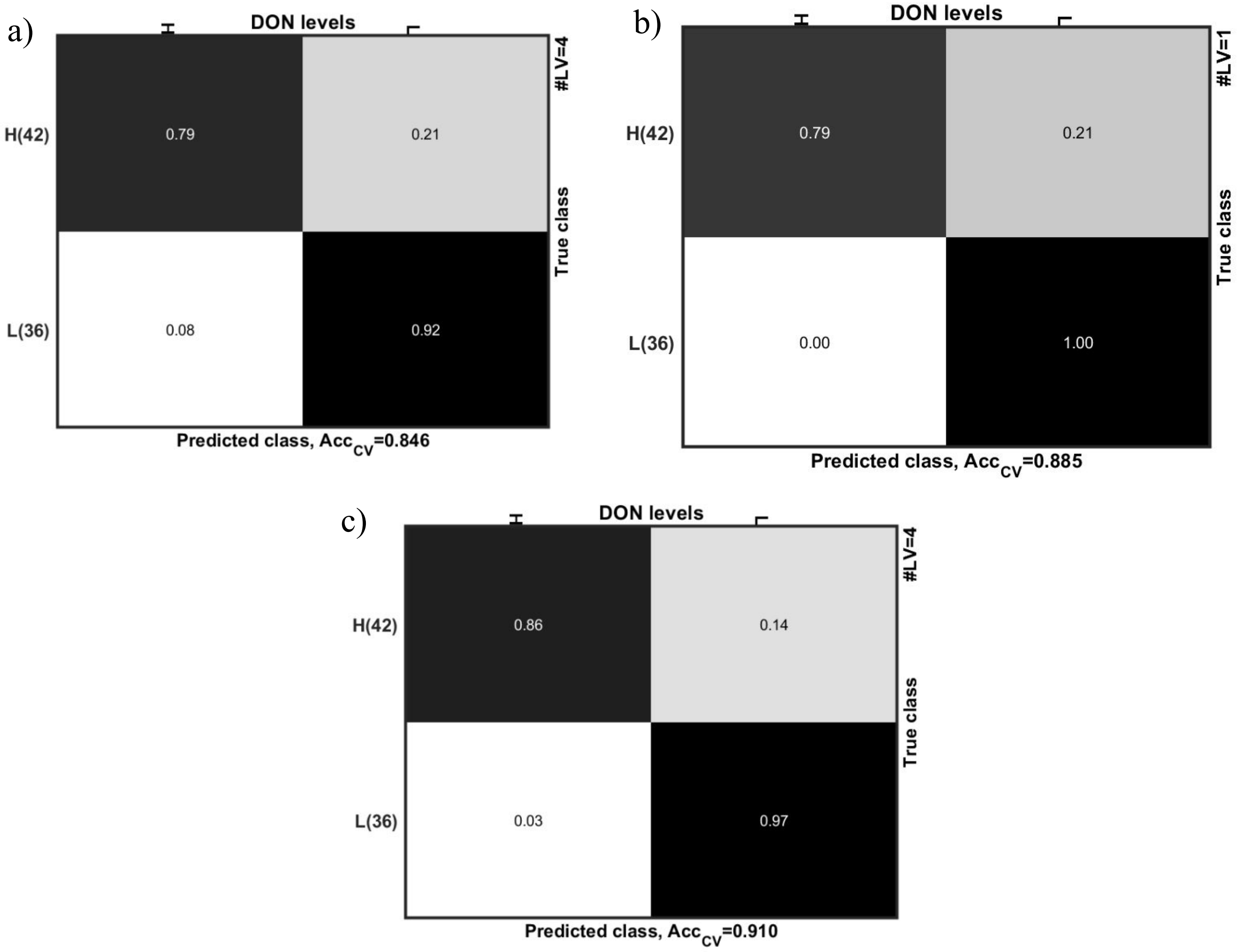

S 1. SPLS-DA classification results for wheat samples into two groups: high (H) DON > 1250 µg/kg and low (L) DON < 1250 µg/kg extracted by different solvents a) methanol:water (30:70) b) ethanol:water (30:70) c) water (100). Acc_{cv} is classification accuracy of the cross validation and LV is the number of latent variables.
